# Correction: Aung et al. Air Permeability, Shock Absorption Ability, and Flexural Strength of 3D-Printed Perforated ABS Polymer Sheets with 3D-Knitted Fabric Cushioning for Sports Face Guard Applications. *Polymers* 2021, *13*, 1879

**DOI:** 10.3390/polym13142280

**Published:** 2021-07-12

**Authors:** Thet Khaing Aung, Hiroshi Churei, Gen Tanabe, Rio Kinjo, Kaito Togawa, Chenyuan Li, Yumi Tsuchida, Phyu Sin Tun, Shwe Hlaing, Hidekazu Takahashi, Toshiaki Ueno

**Affiliations:** 1 Department of Sports Medicine/Dentistry, Graduate School of Medical and Dental Sciences, Tokyo Medical and Dental University, 1-5-45 Yushima, Bunkyo-ku, Tokyo 113-8549, Japan; tka.spmd@tmd.ac.jp (T.K.A.); chu.spmd@tmd.ac.jp (H.C.); gen.spmd@tmd.ac.jp (G.T.); r.kinjo.spmd@tmd.ac.jp (R.K.); kaito321.629@gmail.com (K.T.); licyspmd@tmd.ac.jp (C.L.); 2 Department of Maxillofacial Prosthetics, Graduate School of Medical and Dental Sciences, Tokyo Medical and Dental University, 1-5-45 Yushima, Bunkyo-ku, Tokyo 113-8549, Japan; 3 Department of Oral Biomaterials Engineering, Graduate School of Medical and Dental Sciences, Tokyo Medical and Dental University, 1-5-45 Yushima, Bunkyo-ku, Tokyo 113-8549, Japan; yumi.bmoe@tmd.ac.jp (Y.T.); takahashi.bmoe@tmd.ac.jp (H.T.); 4 Moe Myitta Dental Clinic, No.117, Corner of 26th × 76th Street, Chaayetharsan Township, Mandalay 05024, Myanmar; mgphyusintun@gmail.com; 5 Department of Prosthodontics, The University of Dental Medicine, Mandalay, 62nd Street, Chan Mya Thazi Township, Mandalay 05041, Myanmar; drshlaingdental@gmail.com

**Error in Figures 3 and 6**

In the original article [1], there was a mistake in “**Figure 3. Schematic diagram of setup for shock absorption test.”** as published. **The labels in Figure 3 (cushion material and core material) are in the wrong order.** The corrected “**Figure 3. Schematic diagram of setup for shock absorption test.”** appears below. The authors apologize for any inconvenience caused and state that the scientific conclusions are unaffected. The original article has been updated.

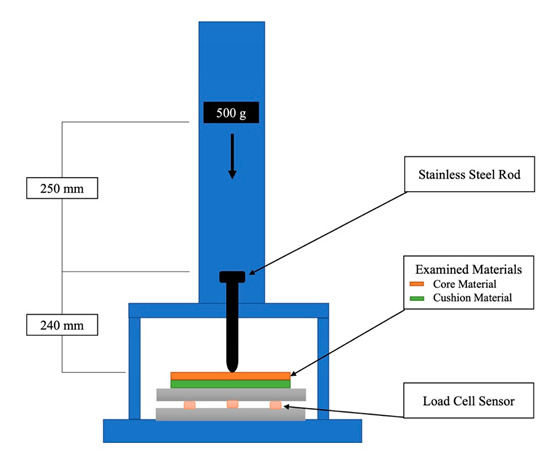



In the original article, there was a mistake in **“Figure 6. Maximum loads of various specimens obtained from the shock absorption tests. The values of the same letter were not significantly different (*p* > 0.05).**” as published. **The label in Figure 6 has a typing error in the first column ABC in place of BCD.** The corrected **“Figure 6. Maximum loads of various specimens obtained from the shock absorption tests. The values of the same letter were not significantly different (*p* > 0.05).”** appears below. The authors apologize for any inconvenience caused and state that the scientific conclusions are unaffected. The original article has been updated.


